# Abnormal Eu^3+^ → Eu^2+^ Reduction in Ca_9−*x*_Mn*_x_*Eu(PO_4_)_7_ Phosphors: Structure and Luminescent Properties

**DOI:** 10.3390/ma16041383

**Published:** 2023-02-07

**Authors:** Elena V. Sipina, Dmitry A. Spassky, Nataliya R. Krutyak, Vladimir A. Morozov, Evgenia S. Zhukovskaya, Alexei A. Belik, Mikhail S. Manylov, Bogdan I. Lazoryak, Dina V. Deyneko

**Affiliations:** 1 Physics Department, Lomonosov Moscow State University, 119991 Moscow, Russia; 2 Skobeltsyn Institute of Nuclear Physics, Lomonosov Moscow State University, 119991 Moscow, Russia; 3 Institute of Physics, University of Tartu, 50411 Tartu, Estonia; 4 Department of Chemistry, Lomonosov Moscow State University, 119991 Moscow, Russia; 5 International Center for Materials Nanoarchitectonics (WPI-MANA), National Institute for Materials Science (NIMS), Tsukuba 305-0044, Ibaraki, Japan; 6 Laboratory of Arctic Mineralogy and Material Sciences, Kola Science Centre, Russian Academy of Sciences, 184209 Apatity, Russia

**Keywords:** TCP structure, whitlockite, phosphates, abnormal reduction, photoluminescence

## Abstract

β-Ca_3_(PO_4_)_2_-type phosphors Ca_9−*x*_Mn*_x_*Eu(PO_4_)_7_ have been synthesized by high-temperature solid-phase reactions. The crystal structure of Ca_8_MnEu(PO_4_)_7_ was characterized by synchrotron X-ray diffraction. The phase transitions, magnetic and photoluminescence (PL) properties were studied. The abnormal reduction Eu^3+^ → Eu^2+^ in air was observed in Ca_9−*x*_Mn*_x_*Eu(PO_4_)_7_ according to PL spectra study and confirmed by X-ray photoelectron spectroscopy (XPS). Eu^3+^ shows partial reduction and coexistence of Eu^2+^/^3+^ states. It reflects in combination of a broad band from the Eu^2+^ 4f^6^5d^1^ → 4f^7^ transition and a series of sharp lines attributed to ^5^D_0_ → ^7^F_J_ transitions of Eu^3+^. Eu^2+^/Eu^3+^ ions are redistributed among two crystal sites, M1 and M3, while Mn^2+^ fully occupies octahedral site M5 in Ca_8_MnEu(PO_4_)_7_. The main emission band was attributed to the ^5^D_0_ → ^7^F_2_ electric dipole transition of Eu^3+^ at 395 nm excitation. The abnormal quenching of Eu^3+^ emission was observed in Ca_9−*x*_Mn*_x_*Eu(PO_4_)_7_ phosphors with doping of the host by Mn^2+^ ions. The phenomena of abnormal reduction and quenching were discussed in detail.

## 1. Introduction

The global search for obtainable phosphors emitting in the red region of the spectrum continues to the present, due to the requirements for creating high-quality light from modern LED illuminators. The aims of red phosphors are to improve the color rendering (CRI) and lower the resulting corelated color temperature (CCT) of the LED package. The main requirements for such phosphors are:

(1) A broad excitation band which can be matched well with the light from the LED chip (usually, at 450–470 nm from the InGaN chip). A number of Eu^3+^-doped inorganic red phosphors have been developed with the narrow emission due to the electric dipole ^5^D_0_ → ^7^F_2_ transition located at 610–630 nm. This transition is dominant in most hosts due to the noncentrosymmetric environment. However, such luminescent materials mismatch the excitation wavelengths from the LED chip since the main excitation band of the Eu^3+^ ion is located obviously at 392–396 nm and has a narrow character;
(2) A narrow emission band in the red region (full width at half-maximum (FWHM) should not exceed 20 nm to reduce radiative losses in the near-IR range) [1]. Moreover, the barycenter of the emission band must not lie beyond 650 nm to minimize wasted emission [2];
(3) A high stability in the environment and high luminous efficiency of radiation (LER). LER values increase with the narrowing of the emission line from red phosphor [1]. At the moment, the luminous efficiency from red phosphors can reach values of 54 lm/W (lumens per watt) [3].

Red-emitting phosphors that mostly meet the technological requirements can be created, for example, based on nitrides [4] or oxynitrides, such as (Ba,Sr)_2_Si_5_N_8_:Eu^2+^ [5], (Ca,Sr)SiAlN_3_:Eu^2+^ [6] or Sr[Li_2_Al_2_O_2_N_2_]:Eu^2+^ [7]. For obtaining such compositions, metal nitrides Si_3_N_4_ and AlN are usually used as raw materials which are air-sensitive and require a combination of high temperatures (up to 1700 °C) with a reducing atmosphere [8].

Phosphors based on hexafluorometallates, such as K_2_SiF_6_:Mn^4+^ [9], for instance, satisfy the requirement to reduce the consumption of rare-earth elements [10] and emit an extremely narrow photoluminescence band (used to increase the brightness of displays) due to intra-configuration 3d-3d transitions of Mn^4+^ ions. However, high-cost Si sources and not environment-friendly hydrofluoric acid are used in the synthesis. Moreover, they are very sensitive to moisture in the air and have long decay times, which limit their application.

According to the above, phosphate-based phosphors are important luminescent materials due to their excellent stability and available synthesis conditions. At the same time, the incorporation of Mn^2+^ ions into the β-Ca_3_(PO_4_)_2_-type (β-TCP) structure phosphates makes it possible to both stabilize the crystal structure [11] and to obtain photoluminescent properties from Mn^2+^ ions [12]. The stabilization of the lattice occurs due to the reduction of geometric stress in the octahedral site during Ca^2+^ → Mn^2+^ substitution since the ionic radii of Mn^2+^ (r_VI_ = 0.83 Å) are less than Ca^2+^ (r_VI_ = 1.00 Å). Mn^2+^-doped phosphates show a broad emission band at 600–750 nm, peaked at 650 nm [12,13,14], which corresponds to the red region of the visible spectrum. Mn^2+^ ions, as an activator, show a wide emission band from the ^4^T_1_(^4^G) → ^6^A_1_(^6^S) transition in the PL spectrum [13]. This emission strongly depends on the crystal field and can shift from green to red color. In an octahedral environment with a strong crystal field, Mn^2+^ ions usually generate red emission. If Mn^2+^ ions are located in a tetrahedral environment with a weak crystal field, green emission could be observed [15,16]. A serious advantage of Mn^2+^ doping into the β-TCP structure is a strong absorption of excitation at 450–480 nm, which is matching with the InGaN blue chip [13].

Co-doping strategy using Eu^2+^/Mn^2+^ [17] or Ce^3+^/Mn^2+^ [18] ions can improve the emission intensity due to the energy transfer processes in comparison to single-doped β-TCP-type hosts. For the CIE color adjustment and white light production, the combinations of rare-earth ions with Mn^2+^ at different concentrations can be used, such as Ce^3+^/Tb^3+^/Mn^2+^ or Eu^2+^/Tb^3+^/Mn^2+^. Moreover, there is a possibility to obtain Mn^2+^ to Eu^3+^ energy transfer for enhancement of red Eu^3+^ emission [15,19,20] while Eu^2+^ to Mn^2+^ energy transfer is commonly observed [21]. Some data on Mn^2+^ and RE/Mn^2+^ (RE—rare-earth element) doped β-TCP phosphors are summarized in Table 1.

The idea of the present research was to combine the emission from Eu^3+^ and Mn^2+^ ions in the stable and easily synthesized host to obtain ideal red phosphor. The structure’s features were studied using synchrotron X-ray diffraction. The abnormal reduction of Eu^3+^ in air was observed according to PL spectra and confirmed by XPS data. Moreover, a strong quenching of Eu^3+^ emission was detected in Ca_9−*x*_Mn*_x_*Eu(PO_4_)_7_, which is the opposite to other Ca_9−*x*_M*_x_*Eu(PO_4_)_7_ phosphates with divalent metals, such as Mg^2+^ or Zn^2+^. The mechanisms of self-reduction and quenching are discussed in detail.

## 2. Materials and Methods

The series of phosphates Ca_9−*x*_Mn*_x_*Eu(PO_4_)_7_ was synthesized by high-temperature solid-state route from simple oxides MnO_2_ (99.9%), Eu_2_O_3_ (99.9%), calcium hydrogen phosphate CaHPO_4_·2H_2_O (99.9%) and calcium carbonate CaCO_3_ (99.9%). The reagents of standard grade were checked for purity and used without further purification. The raw materials were weighted and thoroughly grounded. The syntheses were carried out in air in alundum crucibles at 1100 °C for 50 h. Phase analysis using JCPDS PDF-4 database (ICDD, Newtown Square, PA, USA) revealed that the synthesized samples did not contain any reflections of the initial or impurity phases.

The chemical composition of Ca_8_MnEu(PO_4_)_7_ was determined by energy-dispersive X-ray spectrometry (EDX) using scanning electron microscope (SEM) Tescan VEGA3 (Oxford Instruments, Abingdon, UK) equipped with an Oxford Instruments X-Max 50 silicon drift. The EDX analysis results were based on the Ca_K_, Mn_K_, Eu_L_ and P_K_ edge lines. The oxygen content was not quantified by EDX.

Powder X-ray diffraction (PXRD) patterns were obtained using Thermo ARL X’TRA (Bragg–Brentano geometry, Scintillator detector, CuKα radiation, λ = 1.5418 Å, Thermo Fisher Scientific, Waltham MA, USA). PXRD data were collected in 2θ range from 5° to 75° with 0.02° step at room temperature.

Synchrotron PXRD data for Ca_8_MnEu(PO_4_)_7_ were measured with a large Debye-Scherrer camera (home-made, NIMS, Tsukuba, Japan) at the BL15XU beamline of SPring-8. The intensity data were collected in 2θ range from 1° to 60° with step 0.003°. The incident beam was monochromatized at λ = 0.65298 Å. The samples were packed into Lindemann glass capillaries with an inner diameter of 0.1 mm, which were rotated during the measurement. The absorption coefficients were also measured. Rietveld analysis [27] was performed using JANA2006 software (by Petricek, V., Dusek, M. & Palatinus, L. Institute of Physics, Academy of Science of the Czech Republic, Praha) [28].

The second harmonic generation (SHG) signal was measured with a Q-switched YAG:Nd laser (home-made, Moscow, Russia) at λ_ω_ =1064 nm in the reflection mode. The powder of α-SiO_2_ (3–5 μm particles size) was used as a standard to calibrate the intensity of the SHG signal (*I*_2ω_). The final SHG value is a relation: *I*_2ω_(sample)/*I*_2ω_(SiO_2_).

Differential scanning calorimetry (DSC) measurements were performed on an NETZSCH DSC 204 F1 calorimeter (NETZSCH, Selb, Germany) in the temperature range from 323 to 973 K with heating rate 10 K·min^−1^ in nitrogen flow of 40 mL·min^−1^.

The electrical conductivity, dielectric permittivity ε and dielectric loss tangent tg δ in air were measured by the double-contact method in the frequency range of 1–106 Hz at 300–1270 K (heating rate of 5 K·min^−1^), with the help of precision voltmeter Solartron 7081 (Schlumberger, Houston, TX, USA) and frequency response analyzer Solartron 1260 (Schlumberger, Houston, TX, USA). Ceramic pellet from Ca_8_MnEu(PO_4_)_7_ (1.5 mm thick and 5–6 mm in diameter) was prepared by pressing and sintering at 1473 K for 12 h. Pt paste was put on the flat surfaces of the pellet, and then it was heated at 1023 K for 4 h to produce platinum electrodes.

Magnetic measurements were performed on a SQUID magnetometer (Quantum Design, MPMS-XL-7T, Quantum Design, San Diego, CA, USA) from 400 K to 2 K at an applied field of 10 kOe. Isothermal magnetization measurements, M vs. H, were performed from 70 kOe to 0 Oe at *T* = 2 K.

The surface chemical analysis of Ca_9−*x*_Mn*_x_*Eu(PO_4_)_7_ *x* = 0.2 and *x* = 1.0 phosphates was performed by XPS using a Axis Ultra DLD (Kratos Analytical, Manchester, UK) spectrometer with monochromatic AlK_α_ source X-rays (1486.6 eV). The measurements were performed at pressure better than 5 × 10^−7^ Pa. The area of the surface analyzed was ~300 × 700 μm^2^, which provided statistically reliable average results that represented the general surface of the compact powder. The resolution of the spectrometer measured as the full width at half maximum (FWHM) of the Au4*f*_7/2_ line was about 0.7 eV. The experiments were performed with charge neutralization and use of the C1s level (285.0 eV) arising from the saturated hydrocarbon contamination on the sample surface as the binding energy (E*_b_*, eV) scale reference. Selected region spectra were recorded covering the Ca2s, Ca2p, P2s, P2p, Eu3d, Eu4p, Eu4d, Mn2p, O1s and C1s photoemission peaks. The XPS spectra were measured with an energy step size of 1 eV and a pass energy of 160 eV. The high-resolution XPS spectra were performed with an energy step size of 0.1 eV and a pass energy of 40 eV.

Luminescence excitation spectra and emission spectra under excitation in the UV region were measured using a 150 W xenon lamp (Oriel Instruments, Stratford, CT, USA) as an excitation source, an MDR-206 primary monochromator (Lomo, Saint-Petersburg, Russia) and a LOT-Oriel MS-257 spectrograph (Oriel Instruments, Stratford, CT, USA) equipped with a Marconi CCD detector (Marconi Applied Technologies Limited, Chelmsford, UK). Samples were mounted into a Cryotrade LN-120 vacuum optical cryostat (Cryotrade engineering, Moscow, Russia).

## 3. Results

### 3.1. SEM Observations

**Figure 1:**
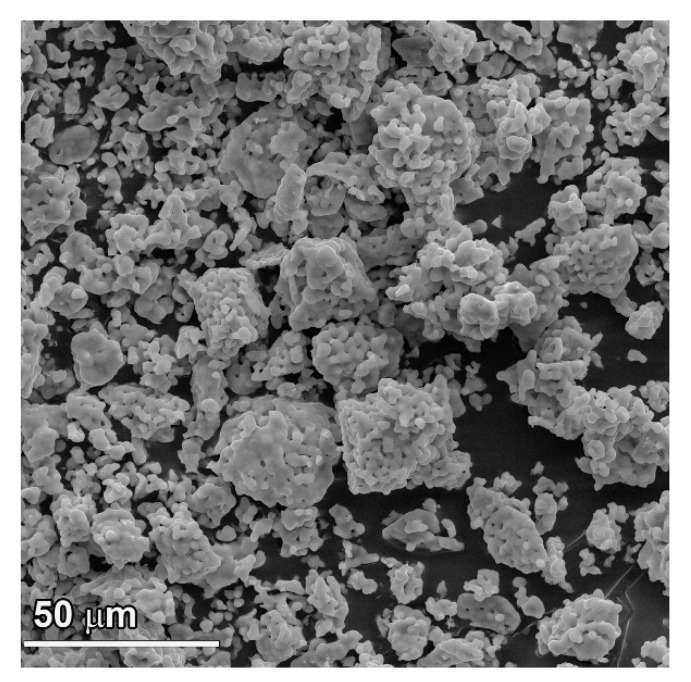
SEM image of Ca_8_MnEu(PO_4_)_7_.

The SEM image of Ca_8_MnEu(PO_4_)_7_ is shown in Figure 1. The sample consists of small particles from 2–5 μm which are slightly agglomerate with each other. According to the EDX data, the ratio between Ca: Mn: Eu: P in Ca_8_MnEu(PO_4_)_7_ was determined as 7.98 ± 0.62: 1.01 ± 0.08: 0.99 ± 0.05: 7.01 ± 0.85. This ratio, defined by EDX data, is close to the expected composition.

### 3.2. SHG, DSC and Dielectric Spectroscopy Measurements

The SHG signal of Ca_8_MnEu(PO_4_)_7_ shows a very weak response. The value of the SHG signal (*I*_2ω_) relative to the quartz standard *I*_2ω_ (SiO_2_) was *I*_2ω_/*I*_2ω_ (SiO_2_) < 0.1, which corresponds to the sensitivity limit of the device. Such a small SGH signal value is attributed to a centrosymmetric structure. Previously studied Ca_8_M^2+^Eu(PO_4_)_7_ phosphates with M^2+^ = Zn^2+^ [29], Mg^2+^ [30], Cd^2+^ [31] showed similar small SGH values, and their structures were defined as centrosymmetric with the space group *R*$\bar{3}$*c*. The studied Ca_8_MnEu(PO_4_)_7_ phosphate complements the group of centrosymmetric β-TCP-type compounds.

For the series of phosphates Ca_9−*x*_Mn*_x_*Eu(PO_4_)_7_, the SHG signal shows a trend to decrease from 0.4 for Ca_9_Eu(PO_4_)_7_ (*x* = 0) to 0 for Ca_8_MnEu(PO_4_)_7_ (*x* = 1). This trend was observed in other β-TCP solid solutions, Ca_9−*x*_Mg*_x_RE*(PO_4_)_7_, *RE* = Dy^3+^ [32], Eu^3+^ [33] and Ca_9−*x*_Zn*_x_RE*(PO_4_)_7_ with *RE* = Tb^3+^ [34], Ho^3+^ [35], Eu^3+^ [29], La^3+^ [36] according to symmetry inhomogeneity of the β-TCP structure.

**Figure 2:**
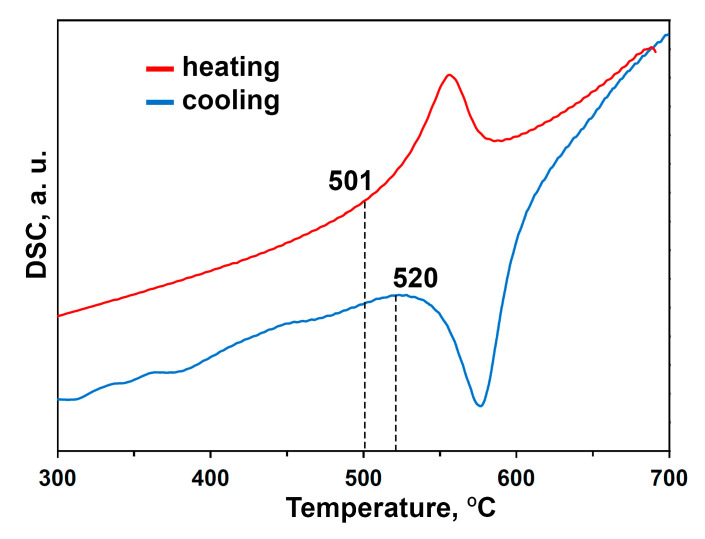
DSC curves in the heating/cooling cycles for Ca_8_MnEu(PO_4_)_7_. The heating/cooling rate is 10 K/min.

The fragments of DSC curves for Ca_8_MnEu(PO_4_)_7_ are showed in Figure 2. DSC curves in the heating and cooling cycles point to the presence of only one peak at 501 and 520 °C, respectively. These peaks are attributed to the first-order phase transition and have a reversible character.

**Figure 3:**
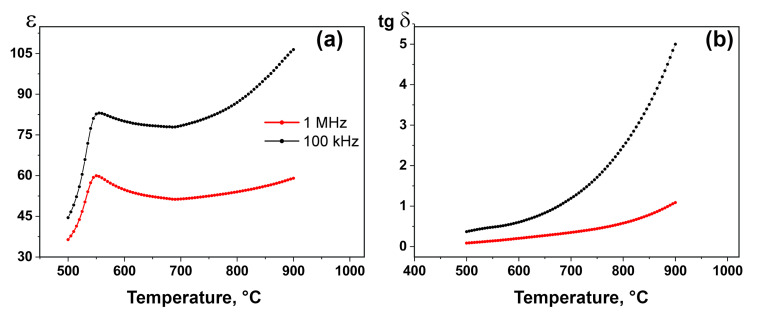
The temperature dependence of the dielectric permittivity ε(T) (**a**) and the dielectric loss tangent (tg δ) (**b**) for Ca_8_MnEu(PO_4_)_7_ at 50 kHz and 1 MHz (on heating).

The temperature dependencies of the dielectric permittivity ε(T) and the dielectric loss tangent tg δ(T) at different frequencies for Ca_8_MnEu(PO_4_)_7_ are shown in Figure 3a and Figure 3b, respectively. ε(T) increases with heating and reaches the phase transition at 525–575 °C with maximum at 550 °C (Figure 3a). A monotonous increase of ε(T) to around Curie temperature (T_c_) seems to be the characteristic behavior. The location of the maximum on the curves does not depend on the frequency (Figure 3a). Such a maximum can characterize both ferroelectric and antiferroelectric phase transitions. However, the absence of an anomaly in tgδ(T) curve at a temperature of 500–1200 °C (Figure 3b) indicates the antiferroelectric character of the phase transition [37,38].

The absence of the SHG signal and the presence of an antiferroelectric phase transition on ε(T) along with effects on DSC curves confirm the centrosymmetric structure of Ca_8_MnEu(PO_4_)_7_. Since the polar and nonpolar space groups *R*3*c* and *R*$\bar{3}$*c* in the β-TCP-type compounds are practically indistinguishable from PXRD data [33], previously it was proved by electron diffraction that Ca_8_MgEu(PO_4_)_7_ [37] crystallizes in the centrosymmetric group *R*$\bar{3}$*c*, and during the phase transition, the symmetry changes from *R*$\bar{3}$*c* to *R*$\bar{3}$*m* [37].

The temperature position of the phase transition in Ca_8_MnEu(PO_4_)_7_ exceeds these values for Ca_8_ZnEu(PO_4_)_7_ (T_c_ ~ 547 °C) [29] and Ca_8_MgEu(PO_4_)_7_ (T_c_ ~ 507 °C) [33]. This fact is due to the difference in the values of the ionic radii of M^2+^ in Ca_8_MEu(PO_4_)_7_. The phase transition occurs at lower temperatures when the smaller ion is placed in the M5 site. Since Mn^2+^ is the largest among these ions (Zn^2+^, Mg^2+^, Mn^2+^), T_c_ shows the biggest value. However, the replacement Ca^2+^ → Mn^2+^ does not significantly affect the phase transition temperature, which is 573 °C for Ca_9_Eu(PO_4_)_7_ [35].

**Figure 4:**
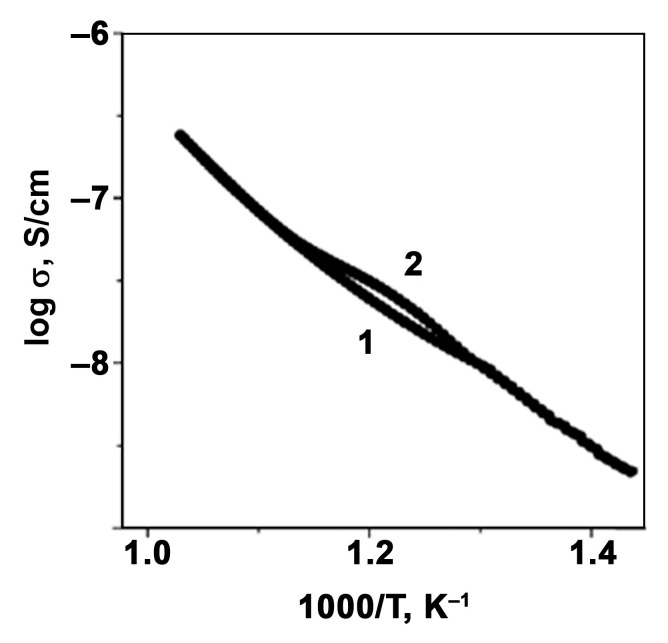
Electric conductivity of Ca_8_MnEu(PO_4_) at 50 kHz on heating (1) and cooling (2).

The temperature dependence of the electric conductivity (σ) of Ca_8_MnEu(PO_4_)_7_ at 50 kHz is shown in Figure 4 in the Arrenius coordinates log(σ)–(10^3^/T). The electroconductivity of Ca_8_MnEu(PO_4_)_7_ is rising with the temperature increasing. The abrupt change in σ at 820–860 K is due to the rearrangement at the antiferroelectric/paraelectric phase transition (*R*$\bar{3}$*c* ↔ *R*$\bar{3}$*m*). Since the conduction temperature during heating is higher than during cooling, such a change in σ also indicates a first-order phase transition. The temperature behavior of the electroconductivity in Ca_8_MnEu(PO_4_)_7_ is similar to other phosphates with the common formula Ca_8_M^2+^*RE*^3+^(PO_4_)_7_ [30,31,39] and is a consequence of the mobility of Ca^2+^ ions [40].

### 3.3. PXRD Study

**Figure 5:**
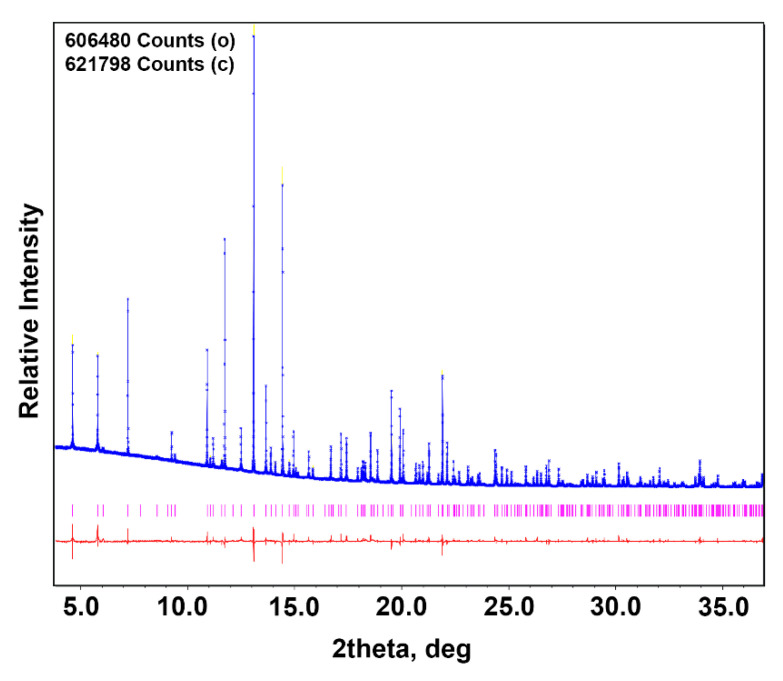
Observed (blue crosses), calculated (blue line) and difference (red line from below) synchrotron PXRD patterns for Ca_8_MnEu(PO_4_)_7_. Magenta bars denote the peak positions of possible Bragg reflections.

PXRD pattern of Ca_8_MnEu(PO_4_)_7_ is similar to other compounds with the β-TCP-type structure (Figure 5). The absence of any impurity reflections on the PXRD pattern shows that Eu^3+^ and Mn^2+^ ions were completely involved in the structure. β-Ca_3_(PO_4_)_2_ structure (sp.gr. *R*3*c*, Z = 6) is a rather rigid structure and consists of isolated tetrahedra PO_4_ that connect CaO_n_ polyhedra into a 3D frame by common vertices [41]. The Ca^2+^ ions are located in sites M1–M5, where M1–M3 and M5 sites are fully occupied, while M4 sites are partly filled and M6 sites are fully vacant.

No vacancies in the anionic sublattice can appear in the β-Ca_3_(PO_4_)_2_-type structure, even during heterovalent substitutions, when [PO4]^3−^ is replaced by [GeO_4_]^4−^ [42] or [SO_4_]^2−^ [43], for instance. Formation of phase-pure phases in these cases requires charge compensation. So, phosphorus atoms fully occupy three (P1, P2 and P3) tetrahedra sites. The symmetry changing *R*3*c* → *R*$\bar{3}$*c* results in an equivalence of M1 and M2 sites in the cationic sublattice and P2O_4_ and P3O_4_ tetrahedra in the anionic sublattice. M5, M3 and P1 sites are located in the center of symmetry, and P1 is in the half-occupied special position 12*c*. Atoms O1 and O2 are located in half-occupied positions 12*c* and 36*f*, respectively.

The atomic coordinates for Ca_8_MgEu(PO_4_)_7_ were used as a starting model for synchrotron data refinement for Ca_8_MnEu(PO_4_)_7_. Manganese ions were refined in the octahedral M5 site, while Eu ions were distributed through M1 and M3 sites with the preference occupation in the M1 site (Table S1 of the Supporting Information). After the structure refinement in an *R*$\bar{3}$*c* model, a good agreement between the calculated and the experimental synchrotron PXRD patterns was observed, as it can be seen from the Figure 5 difference plot. Figure 5 shows fragments of the observed, calculated and difference synchrotron PXRD patterns of Ca_8_MnEu(PO_4_)_7_. Other numerical characteristics showing the quality of the structure refinements are given in Table 2. The fractional atomic coordinates, isotropic atomic displacement parameters and cation occupancies are listed in Table S1 of the Supporting Information. The main interatomic distances are listed in Table S2 of the Supporting Information. CCDC 2237297 contains the supplementary crystallographic data for this paper.

**Figure 6:**
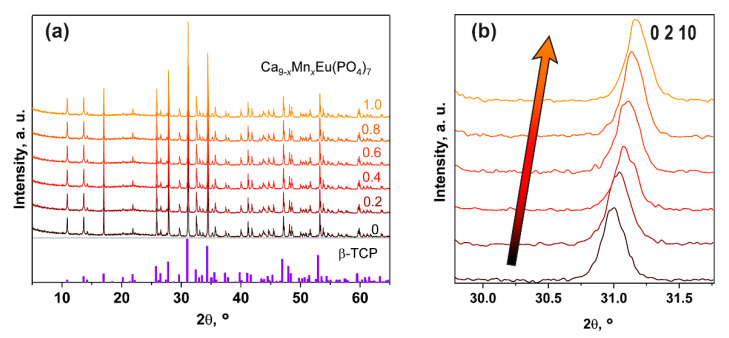
PXRD patterns of Ca_9−*x*_Mn*_x_*Eu(PO_4_)_7_ and the positions of Bragg reflections of β-TCP (β-Ca_3_(PO_4_)_2_, PDF#4 Card No. 00-09-0169) (**a**); The enlarged main reflection (0 2 10) of Ca_9−*x*_Mn*_x_*Eu(PO_4_)_7_ solid solution (**b**).

Figure 6a shows PXRD patterns of Ca_9−*x*_Mn*_x_*Eu(PO_4_)_7_ solid solutions. All the diffraction peaks are matched with β-TCP (PDF#4 Card No. 00-09-0169). Moreover, a shifting of the reflections with the increasing of manganese concentration can be observed (Figure 6b). The reflections move toward larger angles according to Bragg’s rule and difference between the ionic radii of Ca^2+^ (*r*_IV_ = 1.00 Å) and Mn^2+^ (*r*_IV_ = 0.83 Å). The absence of the impurity phases in PXRD patterns and reflections shifting show the successful incorporation of Mn^2+^ ions in the β-TCP structure in all samples.

### 3.4. Magnetic Measurements

**Figure 7:**
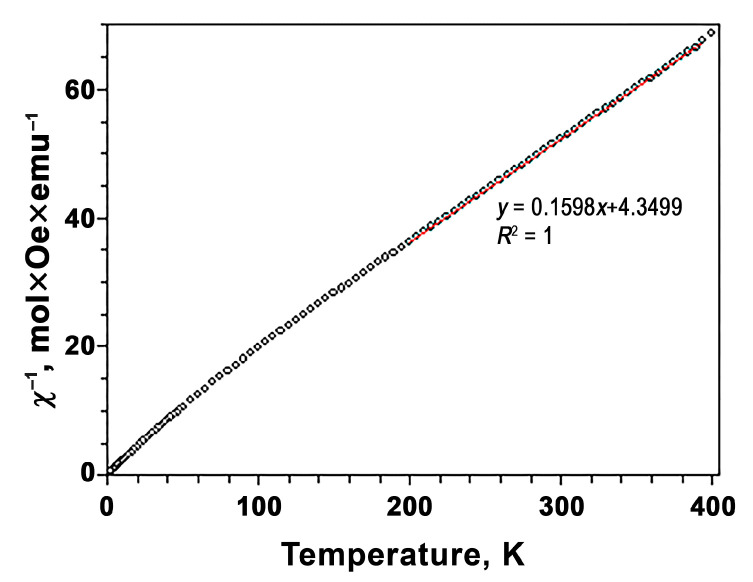
Temperature-dependent inverse magnetic susceptibility curve of Ca_8_MnEu(PO_4_)_7_ at *H* = 10 kOe with fitting results (line).

The inverse magnetic susceptibilities follow the Curie–Weiss law. Between 200 and 395 K, the inverse magnetic susceptibilities are fit by the Curie–Weiss equation:

*χ*(*T*) = *μ*^2^_eff_*N*(3*k*_B_(*T* − *θ*))^−1^,

where *μ*_eff_ is an effective magnetic moment, *N* is Avogadro’s number, *k*_B_ is Boltzmann’s constant and *θ* is the Curie–Weiss temperature. The fitting parameters for Ca_8_MnEu(PO_4_)_7_ were *μ*_eff_ = 7.076(2) (*μ*_B_/f.u.), *μ*_calc_ = 6.823 (*μ*_B_/f.u.) and *θ* = −27.2(2) K. The *μ*_eff_ value was in good agreement with the theoretical value, where *μ*_calc_ is calculated using 3.4*μ*_B_ for Eu^3+^ [44] (Figure 7).

**Figure 8:**
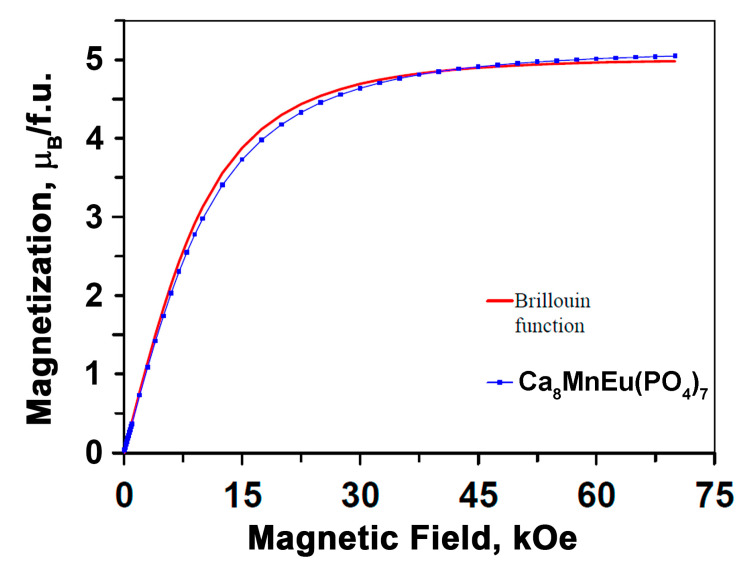
*M* versus *H* curves at *T* = 2 K for Ca_8_MnEu(PO_4_)_7_. The red line shows the Brillouin function with *g* = 2, *S* = 5/2 and *T* = 2 K.

The *M* vs. *H* curve of Ca_8_MnEu(PO_4_)_7_ followed very well the Brillouin function with *g* = 2, *S* = 5/2 and *T* = 2 K, as expected for a free Mn^2+^ cation (Figure 8). The magnetization at 2 K and 70 kOe reached 5.047 (μ_B_/f.u.).

### 3.5. XPS Study

**Figure 9:**
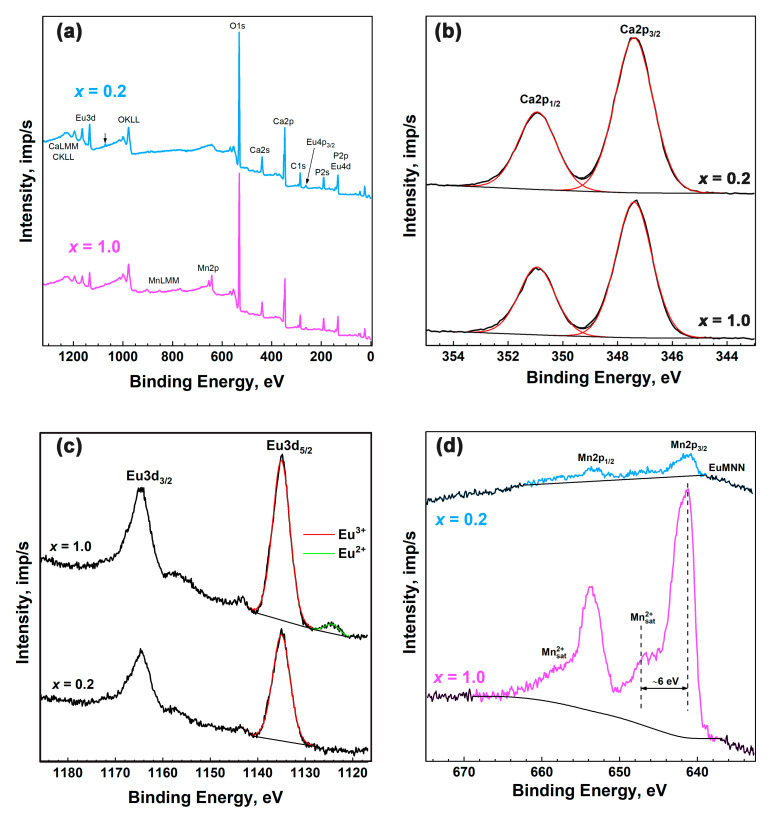
The XPS survey spectrum (**a**) and high-resolution XPS spectra of Ca2p (**b**), Eu3d (**c**) and Mn2p (**d**) peaks of Ca_9−*x*_Mn*_x_*Eu(PO_4_)_7_ with *x* = 0.2 and 1.0.

In the survey XPS scans of Ca_9−*x*_Mn*_x_*Eu(PO_4_)_7_ *x* = 0.2 and *x* = 1.0 samples (Figure 9a), the lines of calcium, europium, manganese, phosphorus, carbon and oxygen were observed. Ca2p XPS spectra (Figure 9b) were used for energy calibration of samples’ spectra to eliminate the charging effect.

In the Eu3d spectra of the samples (Figure 9c), the doublet of lines Eu3d_5/2_ and Eu3d_3/2_ was observed with 1135.0 and 1164.5 eV binding energies. These values are typical for the Eu^3+^ ion [45]. The additional Eu3d_5/2_ component with 1124.4 eV binding energy can be distinguished in the Ca_8_MnEu(PO_4_)_7_ spectrum. This component is attributed to Eu^2+^ [46,47,48,49]. The quantity of divalent europium in Ca_8_MnEu(PO_4_)_7_ is about 5% of the total europium content. At the same time, in [45,50,51], the appearance of such low-energy components is associated with shake-down satellites from the main lines of trivalent europium and indicates that the intensity of such satellites can vary depending on the specific compound of trivalent europium.

**Figure 10:**
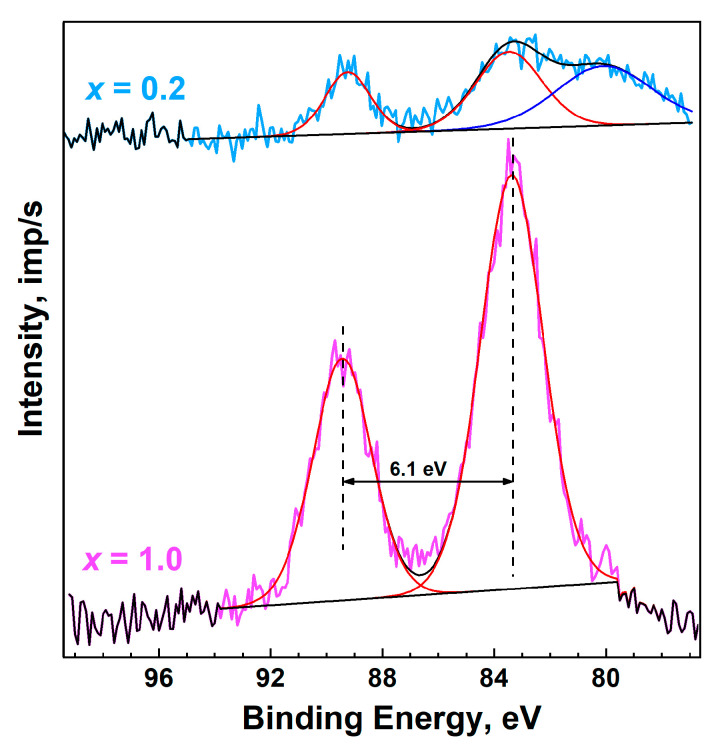
High-resolution XPS spectra of Mn3s peaks of Ca_9−*x*_Mn*_x_*Eu(PO_4_)_7_ *x* = 0.2 and 1.0.

The Mn2p XPS shape of the Ca_8_MnEu(PO_4_)_7_ (Figure 9d) is typical of the divalent state of manganese [52]. The spectrum exhibits pronounced shake-up satellites characteristic of divalent manganese, which are shifted from the main peaks by approximately 6 eV towards higher binding energies. Similarly, in the Mn3s spectrum of Ca_8_MnEu(PO_4_)_7_ (Figure 10), the splitting typical for divalent manganese is observed (Table 2). The Mn2p and Mn3s spectra of Ca_8.8_Mn_0.2_Eu(PO_4_)_7_ are close in shape to Ca_8_MnEu(PO_4_)_7_. However, due to the significantly lower content of manganese in this sample, the spectra are observed to be noticeably noisier. The comparison of the parameters Mn2p and Mn3s spectra with reference data are listed in Table 3.

### 3.6. Photoluminescent Properties

**Figure 11:**
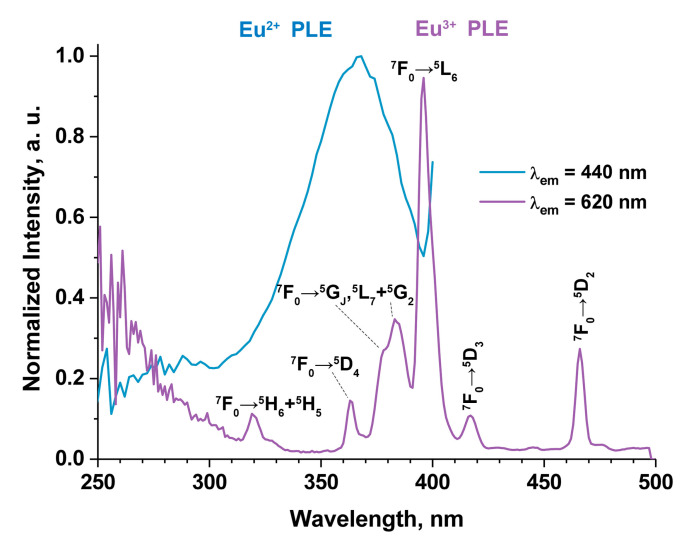
Normalized PLE spectra of Ca_8.2_Mn_0.8_Eu(PO_4_)_7_ at λ_em_ = 440 and 620 nm.

Normalized photoluminescence excitation (PLE) spectra for one of the samples are shown in Figure 11. PLE spectra monitored at 440 nm exhibit an unresolved broad band from 300 to 400 nm, peaking at 365 nm, which originated from the Eu^2+^ 4f-5d-allowed transition (Figure 9). At 620 nm, the PLE spectrum consists of sharp lines attributed to transitions of Eu^3+^ from the ground ^7^F_0_ level to excited levels. The bands are located at 318 nm (^7^F_0_ → ^5^H_3_), 362 nm (^7^F_0_ → ^5^D_4_), 378 nm (^7^F_0_ → ^5^G_J_), 382 nm (^7^F_0_ → ^5^L_7_), 395 nm (^7^F_0_ → ^5^L_6_), 416 nm (^7^F_0_ → ^5^D_3_) and 465 nm (^7^F_0_ → ^5^D_4_), and the area at 250–300 nm is attributed to the charge transfer band (Figure 9). The other samples from the series show the same spectra, and the main difference is in the intensity of the spectra.

**Figure 12:**
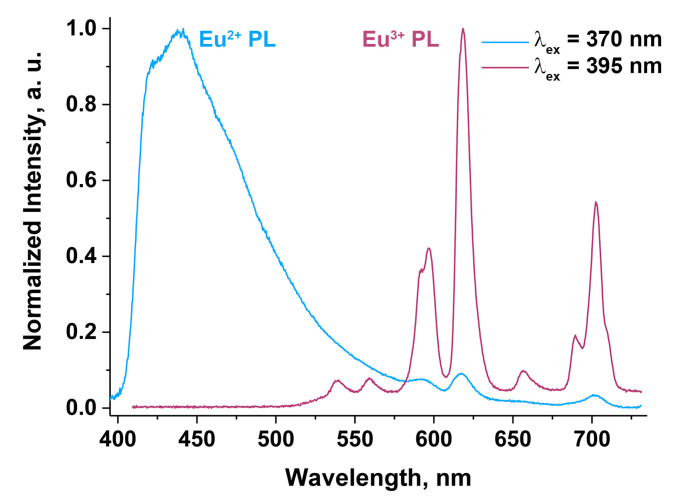
Normalized PL spectra of Ca_9−*x*_Mn*_x_*Eu(PO_4_)_7_ with *x* = 0.8 at λ_ex_ = 370 nm and 395 nm.

Figure 12 shows PL spectra of Ca_8.2_Mn_0.8_Eu(PO_4_)_7_ at different excitation wavelengths. The broad unresolved emission band from 400 to 700 nm appearing under 370 nm excitation can be attributed to the 4*f*^6^5*d*^1^ → 4*f*^7^ transition of Eu^2+^ [57]. The band is asymmetrical and peaked at 440 nm. It arises from different crystallographic sites occupied by Eu atoms in the Ca_8.2_Mn_0.8_Eu(PO_4_)_7_ structure. Since the space group in this sample is *R3c*, there are M1–M3 sites occupied by Eu atoms, and several components in the Eu^2+^ emission can be distinguished [21]. The emission bands from Eu^3+^ are also observed under λ_ex_ = 370 nm; however, their intensity is very low (Figure 12). The location of these lines can be determined at 591 nm (^5^D_0_ → ^7^F_1_), 615 nm (^5^D_0_ → ^7^F_2_), 652 nm (^5^D_0_ → ^7^F_3_) and 698 nm (^5^D_0_ → ^7^F_4_).

Under 395 nm, the excitation PL spectra consist of the typical bands of Eu^3+^ emission (Figure 12). The presence of two types of Eu emission is related to partial abnormal self-reduction in the β-TCP host in agreement with XPS data. The locations of these bands are 593 nm (^5^D_0_ → ^7^F_1_), 618 nm (^5^D_0_ → ^7^F_2_) 655 nm (^5^D_0_ → ^7^F_3_) and 701 nm (^5^D_0_ → ^7^F_4_). The insignificant shifting of the peaks from 370 nm excitation is attributed to poor resolution of Eu^3+^ emission at λ_ex_ = 370 nm. Moreover, the transitions from higher-level ^5^D_1_ to ^7^F_1_ (535 nm) and ^7^F_2_ (555 nm) in terms of ground state can be observed (Figure 12).

**Figure 13:**
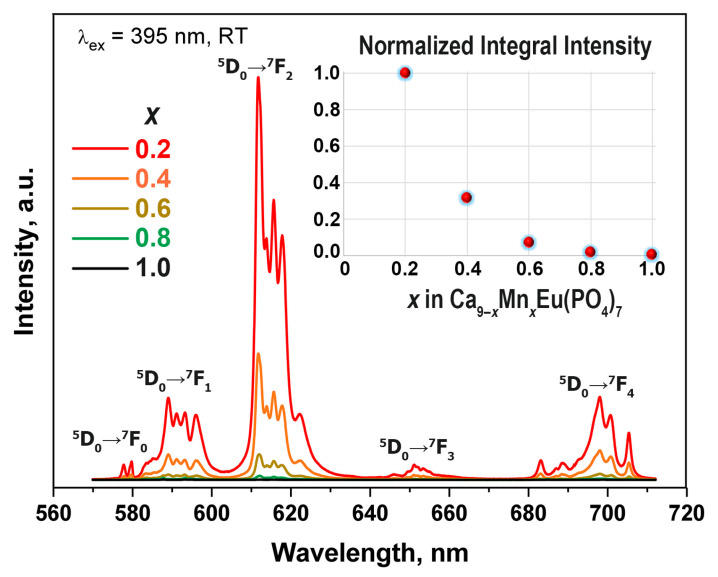
PL spectra for Ca_9−*x*_Mn*_x_*Eu(PO_4_)_7_ at λ_ex_ = 395 nm; the inset shows the dependence of normalized integral intensity on the Mn^2+^ concentration in Ca_9−*x*_Mn*_x_*Eu(PO_4_)_7_.

PL spectra for Ca_9−*x*_Mn*_x_*Eu(PO_4_)_7_ solid solutions at 395 nm excitation with high resolution are shown in Figure 13. The spectra consist of transitions from ^5^D_0_ excited level to ^7^F_0_ (579 nm), ^7^F_1_ (589 nm), ^7^F_2_ (612 nm), ^7^F_3_ (652 nm) and ^7^F_4_ (697 nm) levels. The normalized integral intensity of luminescence can be observed from the inset in Figure 13. It can be seen that PL intensity dramatically decreases with Mn^2+^ doping. This trend contradicts with the other Ca_9−*x*_M*_x_*Eu(PO_4_)_7_ (M = Zn^2+^, Mg^2+^) [29] solid solutions, where changing of the symmetry from polar *R*3*c* to nonpolar *R*$\bar{3}$*c* leads to increasing of the luminescence intensity. However, such behavior of PL intensity in Eu^3+^ and Mn^2+^ co-doped isostructural Ca_3_(VO_4_)_2_ was observed in [58]. The quenching of Eu^3+^ emission by Mn^2+^ doping in the β-TCP host is caused by the energy transfer from Eu^3+^ to Mn^2+^; however, the effective emission from Mn^2+^ ions is absent (Figure 12 and Figure 13). The energy transfer can be relaxed by the ^4^T_1_ energy level of Mn^2+^ and then nonradiative relaxation to the ^6^A_1_ ground state of Mn^2+^ ions. Moreover, the emission from Mn^2+^ can be overlapped with the ^5^D_0_ → ^7^F_3_ transition of Eu^3+^. In addition, the substitution Ca^2+^ → Mn^2+^ is accompanied by the *R*3*c* → *R*$\bar{3}$*c* symmetry changing and the formation of defects in the structure, which may act as quenching centers of photoluminescence.

**Figure 14:**
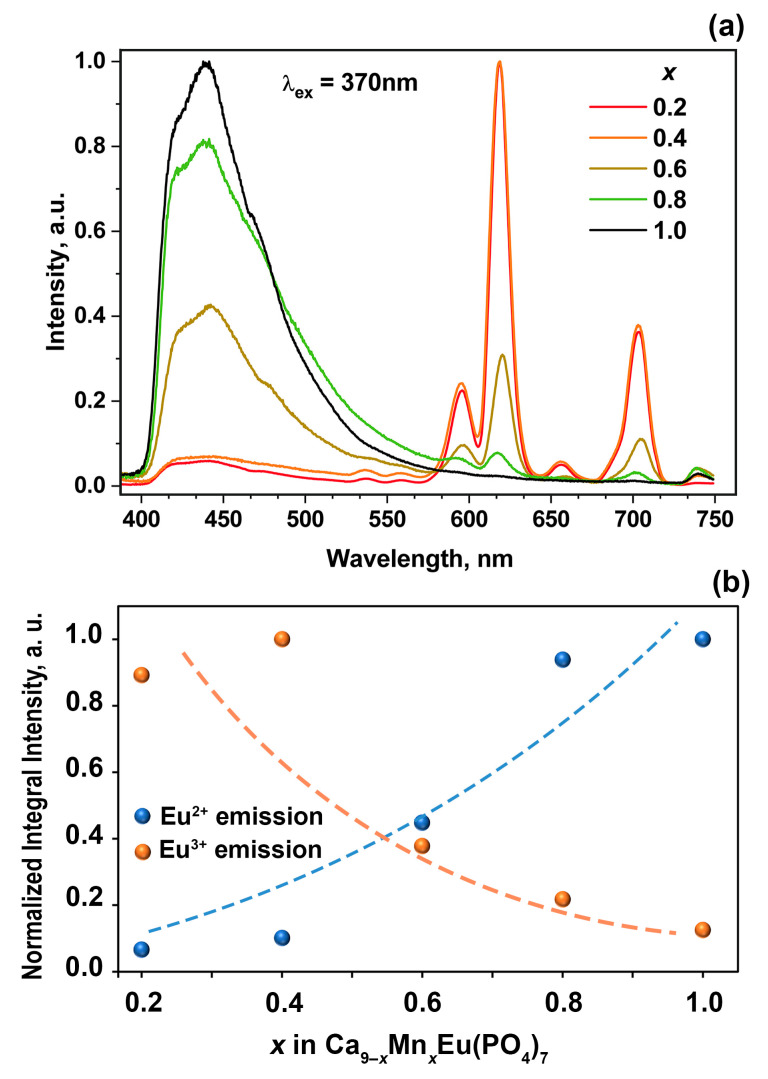
(**a**) PL spectra for Ca_9−*x*_Mn*_x_*Eu(PO_4_)_7_ at λ_ex_ = 370 nm; (**b**) the dependence of normalized integral intensity of Eu^2+^ and Eu^3+^ emission (λ_ex_ = 370 nm).

PL spectra for Ca_9−*x*_Mn*_x_*Eu(PO_4_)_7_ solid solutions at 370 nm excitation are shown in Figure 14a. The intensity of Eu^3+^ emission also decreases with rising of Mn^2+^ concentration. Simultaneously, the intensity of the band attributed to Eu^2+^ emission (at ~450 nm) increases. Such behavior can be clearly observed from the dependence of normalized integral intensity of Eu^2+^ and Eu^3+^ emission on Mn^2+^ concentration (Figure 14b). The position and profile of the Eu^2+^ band do not change with the Mn^2+^ concentration, which points to the invariability of the surrounding crystal field strength. Actually, since Eu and Mn atoms occupy different crystal sites in the β-TCP structure, the environment of Eu does not change. The rising of the Eu^2+^ band intensity (Figure 14a) can be attributed to the increasing of its concentration in the samples. This conclusion also follows from the XPS data.

**Figure 15:**
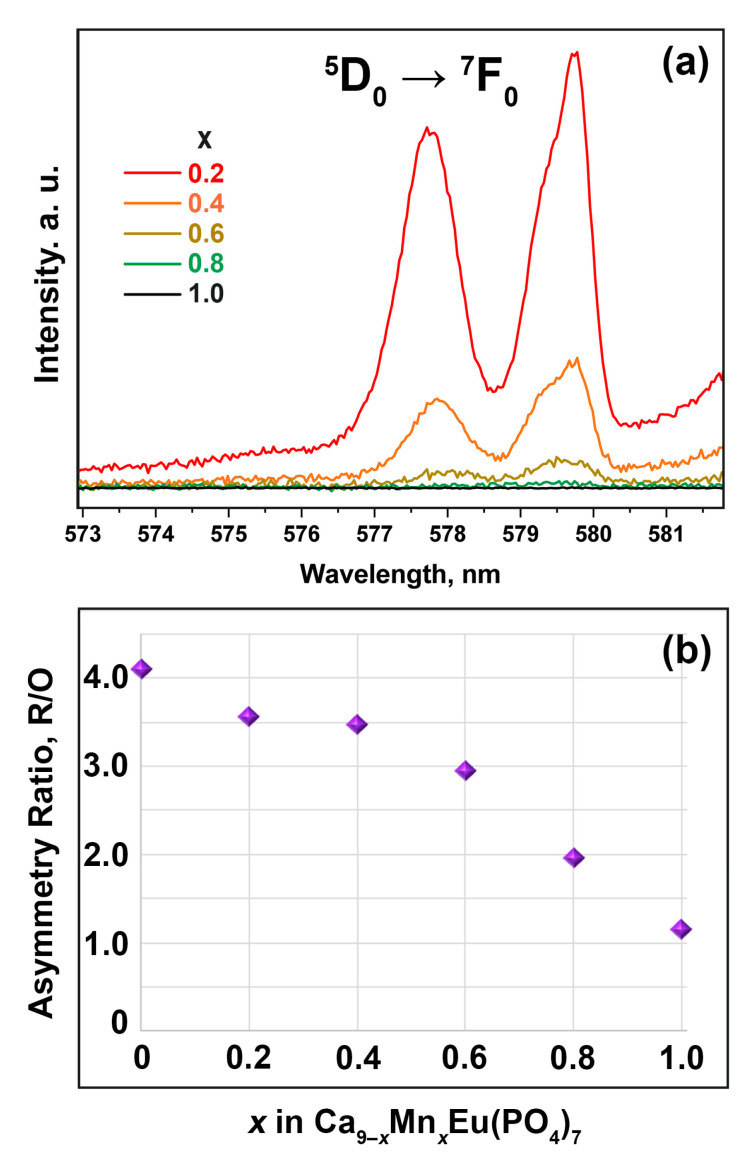
(**a**) ^5^D_0_ → ^7^F_0_ transition in Ca_9−*x*_Mn*_x_*Eu(PO_4_)_7_ at λ_ex_ = 395 nm; (**b**) the dependence of normalized integral intensity of Eu^2+^ and Eu^3+^ emission (λ_ex_ = 370 nm).

To study the evolution of the β-TCP structure in Ca_9−*x*_Mn*_x_*Eu(PO_4_)_7_ solid solutions, the hypersensitive ^5^D_0_ → ^7^F_0_ transition was analyzed. Figure 15 shows an enlarged part of luminescence spectra with the ^5^D_0_ → ^7^F_0_ transition. For the sample with the smallest Mn^2+^ concentration (*x* = 0.2), the presence of nonsymmetrical well-separated bands can be observed (Figure 15a). These peaks reveal the nonequivalent environments of Eu atoms in the structure. The intensity of the ^5^D_0_ → ^7^F_0_ transition decreases with the rising of Mn^2+^ in Ca_9−*x*_Mn*_x_*Eu(PO_4_)_7_ and becomes indistinguishable in samples with *x* = 0.8 and 1.0. According to this, the analysis of the asymmetry ratio (*R*/*O*) can provide the reliable information of the structure’s evolution. *R*/*O* value can be calculated from the observed spectra using the formula [33]:

R/O=∫604 nm638 nmD50→F72∫518 nm604 nmD50→F71

The dependence of *R*/*O* on Mn^2+^ concentration is shown in Figure 15b. The decreasing of *R*/*O* to ~1 for Ca_8_MnEu(PO_4_)_7_ is attributed to the decreasing of the local distortion of the Eu environment in agreement with the structural data.

## 4. Discussion

The abnormal reduction Eu^3+^ → Eu^2+^ in inorganic phosphors prepared using high-temperature solid-state reactions in air was observed in numerous studies [59,60,61]. Usually, this reduction leads to the coexistence of two types of europium oxidation states. There is no information on the full reduction of Eu^3+^ into Eu^2+^ in non-reduction media, so it is difficult to control the efficiency of reduction and luminescence intensity [62]. However, it should be noted that Mn ions in our study were fully reduced from Mn^4+^ (MnO_2_ as initial phase) to the Mn^2+^ state, which was shown by XPS and PL measurements.

The conditions for the abnormal reduction in oxosalts phosphors obtained in air using a high-temperature solid-state reaction were proposed in [63]. These conditions meet the requirements in the β-TCP type host:

(1) There are no oxidizing ions in the structure;
(2) The β-TCP-type host is based on tetrahedral anion groups (PO_4_^3−^);
(3) The doped ions (Eu^3+^ and Mn^4+^) substitute the ions with lower valences (Ca^2+^) in the host;
(4) The substituted cation (Ca^2+^) has ionic radii close to Eu^2+^ (see Table 4 below).

The possibilities of Eu abnormal reduction in the β-TCP-type structure can be explained by the following reasons.

The structures of phosphates with the β-TCP type are built from PO_4_ tetrahedra which connect all of the polyhedra by common oxygen atoms into a 3D network. These O atoms are shared by the adjacent polyhedra and tetrahedra and also lined columns A and B in the β-Ca_3_(PO_4_)_2_ structure. Well chemically bonded O atoms form a rigid structure. This rigid 3D structure of phosphates can shield and isolate the reduced Eu^2+^ and Mn^2+^ ions from the oxidizing attack of oxygen from the atmosphere.

Second, Mn^4+^ may be a luminescent center as well, and its red emission in octahedral sites is due to spin-forbidden ^2^E_g_ → ^4^A_2g_ transitions. However, due to charge imbalance of Mn^4+^ and Ca^2+^, for such substitution, the charge compensation scheme in the anionic part is required [42]:

$$[{\mathrm{PO}}_{4}]3^{-}+\frac{3}{2}{\mathrm{Ca}}^{2+}\;\to \;{\mathrm{Mn}}^{4+}+{\left[{\mathrm{EO}}_{4}\right]}^{4-}+\frac{1}{2}V_{\mathrm{ca}}$$

where *V*_Ca_ is a calcium vacancy, and [EO_4_]^4−^ is an anion with four negative charges, such as GeO_4_^4−^ or SiO_4_^4−^, for instance. Since no charge compensation was applied, Mn^4+^ could transfer to the Mn^2+^ state, which is more suitable for isovalent substitution. Usually, to stabilize manganese in the +2 oxidation state in the β-TCP hosts, MnCO_3_ is used as a raw material in a reduction atmosphere [13,14,62].

Third, there is a size mismatch between Mn^4+^ and Ca^2+^ in the β-TCP host. This mismatch can be estimated by the ionic radius percentage difference (*D_r_*). For isomorphic substitution, this value could not exceed 30%. The calculation of the ionic radius percentage difference *D_r_* can be made by the formula:

$$D_{r}=\frac{|R_{h}\left(CN\right)-R_{d}(CN)|}{R_{h}\left(CN\right)}$$

where dopant *R_d_*(*CN*) and host *R_h_*(*CN*) ions are in the corresponding coordination numbers (*CN*). *D_r_* values for different sites in the β-TCP host are given in Table 4. From the above data, Mn^2+^ doping into the β-TCP host is more preferable. The absence of emission from Mn^4+^ in the octahedra environment (red emission [9,64]) in the studied PL spectra shows its full reduction.

The reduction of Eu^3+^ can be explained by a charge compensation model. According to the difference in the oxidation state of Ca^2+^ and Eu^3+^ ions, two Eu^3+^ substitute three Ca^2+^ to keep the electroneutrality in the β-TCP host. Hence, one vacancy $V_{Ca}^{\prime \prime }$ with two negative charges locates in the M4 site, while two defects of the cation site $Eu_{Ca}^{•}$ with a positive charge in each could be produced:

$$3{\mathrm{Ca}}^{2+}+2{\mathrm{Eu}}^{3+}\;\to \;V_{Ca}^{\prime \prime }+2Eu_{Ca}^{•}$$

In this substitution, $V_{Ca}^{\prime \prime }$ acts as a donor of electrons, while $Eu_{Ca}^{•}$ is an acceptor of electrons. Thus, electrons can be transferred from the vacancy as follows during thermal treatment:

$$V_{Ca}^{\prime \prime }\;\to \;V_{Ca}^{\times }+2e^{–}$$

and the defect Eu^3+^ captured electrons and further reduced Eu^3+^ to Eu^2+^:

$$2Eu_{Ca}^{•}+2e^{–}\;\to \;2Eu_{Ca}^{\times }$$

**Figure 16:**
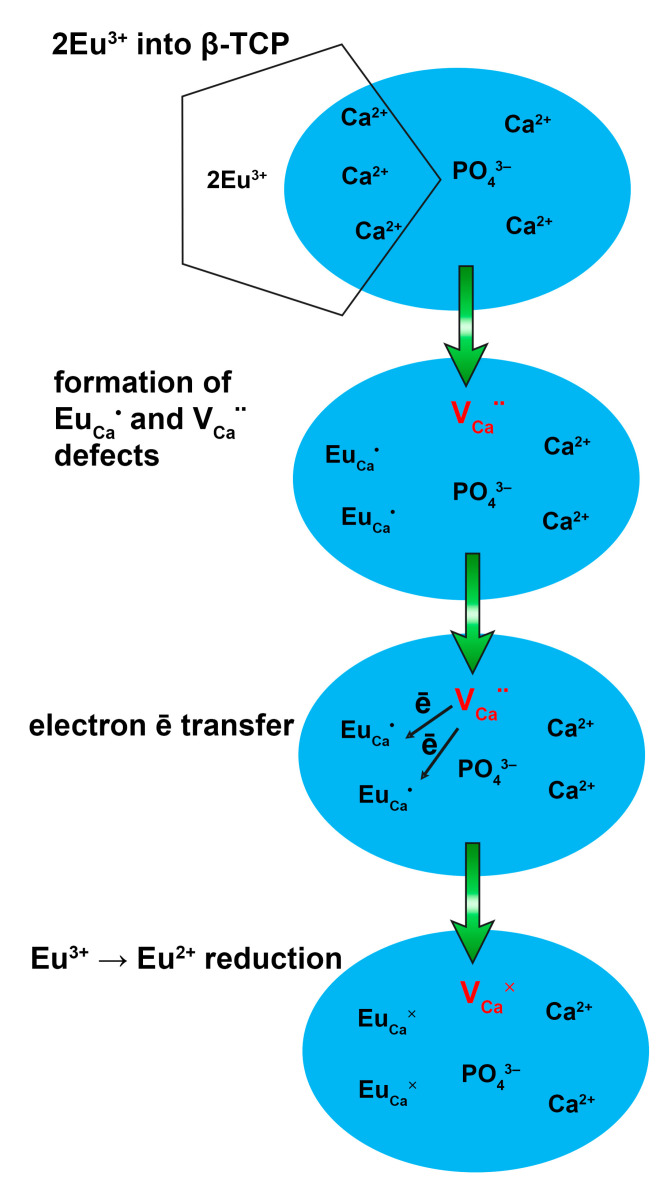
The scheme of abnormal reduction of Eu^3+^ to Eu^2+^ in the β-TCP-type host.

A schematic representation of such reduction is present in Figure 16. A similar mechanism was observed in other β-TCP hosts [65] showing that Eu^3+^ could not be completely reduced to Eu^2+^, even in the reduction atmosphere due to size mismatching.

The reduction of Mn^4+^ can be described as follows. During thermal treatment, interstitial oxygen $O_{i}^{\prime \prime }$ can be formed due to presence of caves along the *c*-axis from PO_4_ frameworks. In order to keep the charge balance, one Mn^4+^ is needed to substitute for two Ca^2+^ ions. So, one vacancy defect $V_{Ca}^{\prime \prime }$ with two negative charges and one $Mn_{Ca}^{\cdot \cdot }$ defect with two positive charges would form. Since MnO_2_ is a raw material, these mechanisms can be ascribed by the following:

$${\mathrm{MnO}}_{2}\;\to \;Mn_{Ca}^{••}+V_{Ca}^{\prime \prime }+O_{0}^{\times }+O_{i}^{\prime \prime }$$

The cause of the full reduction of Mn^4+^ is the possibility of transferring negative charges both from $V_{Ca}^{\prime \prime }$ and interstitial oxygen $O_{i}^{\prime \prime }$ during thermal treatment:

$$V_{Ca}^{\prime \prime }\;\to \;V_{Ca}^{\times }+2e^{–}\;O_{i}^{\prime \prime }\;\to \;O_{0}^{\times }+2e^{–}$$

So, these electrons would be released to reduce Mn^4+^ ions in the Ca^2+^ octahedral M5 site:

$$Mn_{Ca}^{••}+2e^{–}\;\to \;Mn_{Ca}^{\times }$$

Such a reduction was previously observed in oxosalt phosphors [66,67].

## 5. Conclusions

Phosphates Ca_9−*x*_Mn*_x_*Eu(PO_4_)_7_ were obtained by high-temperature solid-phase synthesis. All synthesized samples are isostructural to the β-Ca_3_(PO_4_)_2_. Differential scanning calorimetry and dielectric spectroscopy revealed an antiferroelectric first-order reversible phase transition. The structure of Ca_8_MnEu(PO_4_)_7_ was refined by the Rietveld method (sp. gr. *R*$\bar{3}$*c*) using synchrotron X-ray diffraction. Ca^2+^ and Eu^3+^ ions jointly occupy two sites, M1 and M3, while Mn^2+^ completely occupies the M5 site. Magnetic measurements have shown that Ca_8_MnEu(PO_4_)_7_ contains Mn^2+^ and Eu^3+^ ions. XPS data show the coexistence of europium in +3/+2 oxidation states and manganese in the sole +2 oxidation state. The luminescence of Eu^3+^ and Eu^2+^ ions was found in Ca_9−*x*_Mn*_x_*Eu(PO_4_)_7_. The presence of two types of Eu^2+^/^3+^ emission is associated with the partial abnormal self-reduction of europium in the β-Ca_3_(PO_4_)_2_ matrix. The concentration of Eu^2+^ cations is low (~5% according to XPS) and does not affect the magnetic properties. The intensity of Eu^3+^ emission is dramatically decreased with the rising of Mn^2+^ in Ca_9−*x*_Mn*_x_*Eu(PO_4_)_7_ and attributed to the Eu^3+^ → Mn^2+^ energy transfer. The analysis of the ^5^D_0_ → ^7^F_0_ transition and *R*/*O* values points to the symmetry inhomogeneity (*R*3*c* → *R*$\bar{3}$*c*) in Ca_9−*x*_Mn*_x_*Eu(PO_4_)_7_, such as in other Ca_9−*x*_M*_x_*Eu(PO_4_)_7_ solid solutions with divalent metals.

## Figures and Tables

**Table 1:** The characteristics of PL spectra and some features of Mn^2+^ or RE/Mn^2+^-doped β-TCP-type phosphors.

Host	RE/Mn^2+^ Combination	PL Spectra (Band Location/Peak of the Band)	Features	Ref.
Ca_9_Mn_1−*x*_Na(PO_4_)_7_:*x*M^2+^, M = Zn, Mn	Mn^2+^ and Eu^2+^/Mn^2+^	600–750 nm/655 nm	The co-doping of Zn^2+^/Mg^2+^ and Mn^2+^ broke the intrinsic structural confinement of Mn^2+^ and improved its red emission.	[13]
Sr_19_(Mg_1−x_Mn_x_)_2_(PO_4_)_14_: *y*Eu^2+^	Eu^2+^/Mn^2+^	550–750 nm/610 nm	The emission bands were attributed to Eu^2+^ and Mn^2+^ in different sites.	[17]
Ca_8_MgGd(PO_4_)_7_:Eu^2+^/Mn^2+^	Eu^2+^/Mn^2+^	600–750 nm/650 nm λ_ex_ = 365 nm	Efficient Eu^2+^ → Mn^2+^ energy transfer was observed, Eu^2+^ emission intensity decreased.	[22]
Ca_9_MMn(PO_4_)_7_ (M = Li, Na, K)	Mn^2+^	580–720 nm/645 nm	The excitation by β-source ^90^Sr-^90^Y. Mn^2+^ occupies M5 site, M^+^ ions (Li, Na, K) are located in M4 site.	[23]
Ca_8.82−z_Ga(PO_4_)_7_:0.18Ce^3+^, *z*Mn^2+^	Ce^3+^/Mn^2+^	Two broad emission bands: 350–450 nm/380 nm (Ce^3+^ emission) 600–700 nm/625 nm (Mn^2+^ emission)	The concentration quenching was observed above 9 mol. % Mn^2+^; the decreasing of quantum yield from 62.3% to 67 % with increasing Mn^2+^ concentration was explained by energy loss during Ce^3+^ → Mn^2+^ energy transfer process.	[24]
Ca_10_K(PO_4_)_7_:Eu^2+^, Mn^2+^	Eu^2+^/Mn^2+^	Two broad emission bands (λ_ex_ = 347 nm): 425–500 nm/467 nm (Eu^2+^ emission) 600–700 nm/634 nm (Mn^2+^ emission)	Decreasing of PL intensity with increasing of Mn^2+^ concentration. The concentration quenching was observed above 7 mol. % Mn^2+^; resonant type of Eu^2+^ → Mn^2+^ energy transfer process via a dipole–quadrupole mechanism.	[14]
Ca_9_MgK(PO_4_)_7_:Eu^2+^, Mn^2+^	Eu^2+^/Mn^2+^	Two broad emission bands (λ_ex_ = 347 nm) 425–500 nm/467 nm (Eu^2+^ emission) 600–700 nm/634 nm (Mn^2+^ emission)	Resonant type of Eu^2+^ → Mn^2+^ energy transfer with mechanism via a dipole–quadrupole interaction.	[25]
Ca_8_ZnCe(PO_4_)_7_:Eu^2+^, Mn^2+^	Ce^3+^/Eu^2+^/Mn^2+^	Three broad bands peaked (λ_ex_ = 285 nm) 320–420 nm/375 nm (Ce^3+^ emission) 450–575 nm/500 nm (Eu^2+^ emission) 580–700 nm/645 nm (Mn^2+^ emission)	The energy transfers of Ce^3+^→Eu^2+^/Mn^2+^ and Eu^2+^ → Mn^2+^ ions were investigated. The emitting color can be adjusted from violet-blue to green/red-orange/white by doping/co-doping.	[26]

**Table 2:** Crystallographic data for Ca_8_MnEu(PO_4_)_7_ (SG *R*$\bar{3}$*c*, Z = 6, T = 293 K).

Sample Composition	Ca_8_MnEu(PO_4_)_7_
Lattice parameters: *a*, Å	10.39826(1)
*c*, Å	37.17350(5)
Unit cell volume *V*, Å^3^	3480.851(7)
Calculated density, g/cm^3^	3.413
Data Collection:	
Diffractometer	BL15XU beamline of SPring-8
Radiation/Wavelength (λ, Å)	Synchrotron/0.65298
Absorption coefficient, μ (mm^−1^)	4.381
F(000)	3462
2θ range (°)	2.040–60.237
Step scan (2θ)	0.003
*I*_max_	606,480
Number of points	19,391
Refinement:	
Refinement	Rietveld
Background function	Legendre polynoms, 15 terms
No. of reflections (all/observed)	945/922
No. of refined parameters/refined atomic parameters	43/34
*R* and *R*_w_ (%) for Bragg reflections (*R*_all_/*R*_obs_)	5.29/5.95 and 5.05/4.81
R_P_ and R_wP_; R_exp_	2.06, 3.31, 0.63
Goodness of fit (ChiQ)	5.25
Max./min. residual density(e) (Å^3^)	0.65/−0.88

**Table 3:** Relative position of the satellite (ΔMn2p_sat_) in Mn2p XPS spectra and splitting (ΔMn3s) Mn3s XPS spectra of the studied Ca_9−*x*_Mn*_x_*Eu(PO_4_)_7_ (*x* = 0.2 and 1.0) samples and reference manganese oxides, eV.

Sample	ΔMn2p_sat_	ΔMn3s	Reference
Ca_8_MnEu(PO_4_)_7_	~6.0	6.1	This work
Ca_8.8_Mn_0.2_Eu(PO_4_)_7_	~6.0	5.8	This work
MnO	~6.0	6.1	[53,54]
Mn_2_O_3_	10.1	5.4	[55]
MnO_2_	11.8	4.4	[56]

**Table 4:** Calculated *D_r_* values for Mn and Eu in different oxidation states in the β-TCP host.

Site	*D_r_* Value, %, for Doped Ion			
	Mn^2+^	Mn^4+^	Eu^2+^	Eu^3+^
Ca1–Ca3 *CN*8 (r =1.12 Å)	14 (*r* = 0.96 Å)	-	11 (*r* = 1.25 Å)	4 (*r* = 1.07 Å)
Ca5 *CN*6 (r =1.00 Å)	17 (*r* = 0.83 Å)	47 (*r* = 0.53 Å)	17 (*r* = 1.17 Å)	5 (*r* = 0.95 Å)

*CN* is a coordination number; *r* is the ionic radii in the corresponding *CN*.

## Data Availability

Data will be available from the corresponding author on reasonable request.

## Supplementary Materials

The following supporting information can be downloaded at: https://www.mdpi.com/article/10.3390/ma16041383/s1, Table S1: Atomic coordinates, displacement parameters (Å^2^) and site-occupancy factors (SOFs) in the structure of Ca_8_MnEu(PO_4_)_7_; Table S2: Selected interatomic distances for Ca_8_MnEu(PO_4_)_7_.
