# Supplementary material for: Abnormal Eu3+ → Eu2+ Reduction in Ca9−xMnxEu(PO4)7 Phosphors: Structure and Luminescent Properties
Source: Materials (Basel). 2023 Feb 7;16(4):1383. doi: 10.3390/ma16041383 (PMC9962388; doi:10.3390/ma16041383)
Supplement: Supplementary file 1 [file materials-16-01383-s001.zip › materials-2175993-supplementary.pdf]

Abnormal $Eu^{3+} \rightarrow Eu^{2+}$ Reduction in $Ca_{9-x}Mn_xEu(PO_4)_7$ Phosphors: Structure and Luminescent Properties

Elena V. Sipina [1], Dmitry A. Spassky [2,3], Nataliya R. Krutyak [1,3], Vladimir A. Morozov [4], Evgenia S. Zhukovskaya [4], Alexei A. Belik [5], Mikhail S. Manylov [4], Bogdan I. Lazoryak [4] and Dina V. Deyneko [4,6,*]

[1] Physics Department, Lomonosov Moscow State University, 119991 Moscow, Russia
[2] Skobeltsyn Institute of Nuclear Physics, Lomonosov Moscow State University, 119991 Moscow, Russia
[3] Institute of Physics, University of Tartu, 50411 Tartu, Estonia
[4] Department of Chemistry, Lomonosov Moscow State University, 119991 Moscow, Russia
[5] International Center for Materials Nanoarchitectonics (WPI-MANA), National Institute for Materials Science (NIMS), Tsukuba 305-0044, Ibaraki, Japan
[6] Laboratory of Arctic Mineralogy and Material Sciences, Kola Science Centre, Russian Academy of Sciences, 184209 Apatity, Russia
* Correspondence: deynekomsu@gmail.com

Table S1. Atomic coordinates, displacement parameters (Å^2) and site-occupancy factors (SOFs) in the structure of $Ca_8MnEu(PO_4)_7$.

Atom	Wyckoff site	*x*	*y*	*z*	U_{iso}	SOF
M1	36	0.7210(1)	0.8513(2)	0.43391(3)	0.0029(3)	$Ca_{0.884}$+$Eu_{0.116}$
M3	36	0.1292(4)	0.2752(3)	0.32625(6)	0.0209(1)	$Ca_{0.449}$+$Eu_{0.051}$
M5	6	0	0	0	0.0144(9)	$Mn_{1.000}$
P1	12	0	0	0.2686(2)	0.016(2)	$P_{0.500}$
P2	36	0.3189(2)	0.1752(3)	0.36531(6)	0.0054(6)	P_1
O1	12	0	0	0.3131(4)	0.0097	$O_{0.5}$
O2	36	0.0044(5)	-0.1416(1)	0.2577(3)	0.0097	$O_{0.5}$
O3	36	0.2873(4)	0.1717(5)	0.3256(2)	0.0058	O_1
O4	36	0.2480(5)	0.0155(5)	0.382445	0.0058	O_1
O5	36	0.2694(5)	0.2802(5)	0.3819(4)	0.0058	O_1
O6	36	0.4917(4)	0.2484(7)	0.3717(1)	0.0058	O_1

Table S2. Selected interatomic distances for $Ca_8MnEu(PO_4)_7$.

Polyhedra	Distance	Value, Å
$M1O_8$	*M*1-O2	2.431(2)
	*M*1-O2	2.358(7)
	*M*1-O3	2.355(3)
	*M*1-O4	2.348(5)
	*M*1-O4	2.707(5)
	*M*1-O5	2.403(5)
	*M*1-O5	2.579(6)
	*M*1-O6	2.404(6)
	*M*1-O6	2.458(4)
	**<*M*1-O>**	**2.449**
$M3O_9$	*M*3-O1	2.543(2)
	*M*3-O2	2.920(1)
	*M*3-O3	2.383(5)
	*M*3-O3	2.709(4)
	*M*3-O3	2.882(5)
	*M*3-O4	2.479(5)
	*M*3-O4	2.571(4)
	*M*3-O5	2.533(5)
	*M*3-O5	2.242(6)
	**<*M*3-O>**	**2.585**
$M5O_6$	*M*5-O6×6	2.132(3)
	**<*M*5-O>**	**2.132**
$P1O_4$	P1-O1	1.577(5)
	P1-O2×3	1.555(1)
	**<P1-O>**	**1.561**
$P2O_4$	P2-O3	1.510(4)
	P2-O4	1.568(4)
	P2-O5	1.546(5)
	P2-O6	1.574(3)
	**<P2-O>**	**1.550**
